# A Qualitative Analysis of Factors Influencing HPV Vaccine Uptake in Soweto, South Africa among Adolescents and Their Caregivers

**DOI:** 10.1371/journal.pone.0072094

**Published:** 2013-08-30

**Authors:** Ingrid T. Katz, Busisiwe Nkala, Janan Dietrich, Melissa Wallace, Linda-Gail Bekker, Kathryn Pollenz, Laura M. Bogart, Alexi A. Wright, Alexander C. Tsai, David R. Bangsberg, Glenda E. Gray

**Affiliations:** 1 Brigham and Women’s Hospital, Boston, Massachusetts, United States of America; 2 Harvard Medical School, Boston, Massachusetts, United States of America; 3 Massachusetts General Hospital Center for Global Health, Boston, Massachusetts, United States of America; 4 Perinatal HIV Research Unit, University of the Witwatersrand, Johannesburg, South Africa; 5 Desmond Tutu HIV Centre, Institute of Infectious Diseases and Molecular Medicine, University of Cape Town, Cape Town, South Africa; 6 Children’s Hospital Boston, Boston, Massachusetts, United States of America; 7 Dana Farber Cancer Institute, Boston, Massachusetts, United States of America; 8 Ragon Institute of Massachusetts General Hospital, Massachusetts Institute of Technology, and Harvard, Boston, Massachusetts, United States of America; Fundacion Huesped, Argentina

## Abstract

**Background:**

In South Africa, the prevalence of oncogenic Human Papillomavirus (HPV) may be as high as 64%, and cervical cancer is the leading cause of cancer-related death among women. The development of efficacious prophylactic vaccines has provided an opportunity for primary prevention. Given the importance of psycho-social forces in vaccine uptake, we sought to elucidate factors influencing HPV vaccination among a sample of low-income South African adolescents receiving the vaccine for the first time in Soweto.

**Methods:**

The HPV vaccine was introduced to adolescents in low-income townships throughout South Africa as part of a nationwide trial to understand adolescent involvement in future vaccine research targeting human immunodeficiency virus (HIV). We performed in-depth semi-structured interviews with purposively-sampled adolescents and their care providers to understand what forces shaped HPV vaccine uptake. Interviews were recorded, transcribed, translated, and examined using thematic analysis.

**Results:**

Of 224 adolescents recruited, 201 initiated the vaccine; 192 (95.5%) received a second immunization; and 164 (81.6%) completed three doses. In our qualitative study of 39 adolescent-caregiver dyads, we found that factors driving vaccine uptake reflected a socio-cultural backdrop of high HIV endemnicity, sexual violence, poverty, and an abundance of female-headed households. Adolescents exercised a high level of autonomy and often initiated decision-making. Healthcare providers and peers provided support and guidance that was absent at home. The impact of the HIV epidemic on decision-making was substantial, leading participants to mistakenly conflate HPV and HIV.

**Conclusions:**

In a setting of perceived rampant sexual violence and epidemic levels of HIV, adolescents and caregivers sought to decrease harm by seeking a vaccine targeting a sexually transmitted infection (STI). Despite careful consenting, there was confusion regarding the vaccine’s target. Future interventions promoting STI vaccines will need to provide substantial information for participants, particularly adolescents who may exercise a significant level of autonomy in decision-making.

## Introduction

Infection with oncogenic Human Papilloma Virus (HPV) is a necessary precursor for invasive cervical carcinoma (ICC) [1]. HPV and Human Immunodeficiency Virus (HIV) work synergistically to increase the malignant potential of dysplastic cervical lesions [2], [3], [4]. South Africa has more HIV-positive citizens than any country in the world, with nearly 3 million women ages 15 and older currently living with HIV [5], and up to two-thirds have concomitant oncogenic HPV infections [6], [7], [8]. HIV-positive women are nearly five times more likely to have high-risk HPV-infection compared to HIV-negative women, leading to ICC becoming the most common cancer-killer among South African women [9], [10], [11], [12].

Cytology-based screening programs have been the primary tool for preventing cervical cancer in nations with abundant resources, however, fewer than 5% of women in low-income nations have had a single Pap smear, and even fewer have had access to more advanced interventions such as colposcopy, biopsy, and curettage [13], [14]. Despite the development of efficacious vaccines to prevent HPV [15], [16], [17], [18], the international movement to expand access for girls and women living in resource-limited settings has been slow. Two HPV vaccines are currently available in South Africa (Gardasil™ and Cervarix™), however, these vaccines have only remained affordable to the roughly 20–25% of the population who have had access to private medical insurance [19]. This has left 75–80% of the population who are most at risk for HPV acquisition, and subsequent morbidity and mortality, unable to access the vaccine.

The government has recently announced that it will begin to provide free HPV vaccines to roughly 500,000 low-income nine- and ten-year-old girls through the public sector in February, 2014 [20]. While increasing distribution of the vaccine remains a focus in South Africa, research from other settings suggests that simply ensuring widespread vaccine availability does not always translate into broad uptake. In nations such as the United States, with abundant resources, only half of those who initiate the vaccine ultimately complete the series [21], [22]. Research focused on the multi-dimensional nature of perceived barriers, including vaccine expense, concerns about adverse effects, discomfort from the injection, and low perceived need for the vaccine [23], has provided a framework to understand why vaccine efficacy does not always translate into vaccine effectiveness, even in settings where the vaccine is broadly available [24].

A unique challenge of the HPV vaccine is that in order to obtain maximum effectiveness, the vaccine needs to be administered prior to an adolescent’s sexual debut [25]. This ultimately requires understanding decision-making for a young adolescent to receive a vaccine targeting a sexually transmitted infection (STI). Given the fact that vaccinating pre-adolescents and adolescents is a relatively new phenomenon in many resource-limited settings, formative socio-behavioral research is essential for providing a framework for optimizing vaccine uptake. Qualitative research in Peru [26], India [27], Uganda [28], and Vietnam [29], [30], illustrate the need to understand psychosocial barriers to HPV vaccination, including concerns about vaccine safety and efficacy, and its impact on future fertility in order to effectively design programs that would optimize vaccine uptake [31].

In South Africa, prior qualitative research with health care providers and policy-makers identified concerns regarding how to promote an STI-vaccine to adolescents and their parents [12]. Additional formative qualitative work has been performed with South African traditional healers [32], and mothers of adolescents [33] to assess knowledge, attitudes, and beliefs about HPV and cervical cancer prevention. These studies have consistently found that participants had limited knowledge of the link between cervical cancer and HPV. Despite this, caregivers (who were primarily identified as mothers) appeared willing to vaccinate their children. An additional finding from these prior studies that appears to be unique to the South African context is the concern raised by participants regarding a child’s risk for rape, and the fact that access to the HPV vaccine could reduce their risk of HPV acquisition if they were forced to have unprotected sex. Community-based participatory research in South Africa has also highlighted participants’ concerns about the impact of sexual violence and poverty on reproductive health in general, beyond cervical cancer prevention [34]. Given the importance of psychosocial factors in vaccine uptake in nations where it has been introduced, we sought to elucidate factors influencing HPV vaccine uptake among a sample of low-income South African adolescents receiving the vaccine for the first time in the context of trial modeling uptake of a future HIV vaccine.

## Methods

### Study Design and Overview

We performed a qualitative study based at Kganya Motsha Adolescent Centre in Soweto, an urban area of 1.3 million people in South Africa, with one of the highest rates of HIV transmission among adolescents worldwide (15–20 year olds are at a 1–2% risk of acquiring HIV annually) [35]. This clinic, funded through the United States Agency for International Development (USAID) and the President’s Emergency Plan for AIDS Relief (PEPFAR) until 2012, opened in 2008 and has provided adolescents with free reproductive health services. Kganya Motsha was selected as one of 6 clinics in low-income townships throughout South Africa to distribute the HPV vaccine, Gardasil™, between April and September in 2010 to adolescent boys and girls ages 12–19 as part of an unpublished multi-centre observational cohort study to model acceptability for a future HIV-vaccine [36]. Those who accepted the vaccine were offered Gardasil™ at 0, 2, and 6 months at no-cost. Participants for the parent study were recruited from neighborhood schools and community outreach and had a right to refuse the vaccine at any stage of the study. Participants were simultaneously offered other preventive interventions for STIs. Participants were required to be HIV-negative, and, for female participants, not pregnant.

### Ethics

All participants provided written informed consent to participate in the parent study. An institutional review board (IRB) approved the parent trial protocol at all participating universities, including the primary site, University of Cape Town. Additional IRB approval was granted for the qualitative phase of this work at University of Witwatersrand and Partners Human Research Committee. Quantitative assessments were performed upon completion of the informed consent process to verify that all participants were adequately informed regarding the HPV vaccine. To enroll in our qualitative study, all adolescents signed an assent form, and had a caregiver sign an informed consent release; adolescents less than 18 years old who were without an adult caregiver were excluded. Consent forms were available in English, IsiZulu, and Sesotho. All participants were informed that participation in the study was voluntary and would not prevent them from receiving the HPV vaccine. South African research staff trained in qualitative research methods collected interview data. The caregiver and adolescent were interviewed separately in private settings to protect confidentiality.

### Data Collection

**Figure 1:**
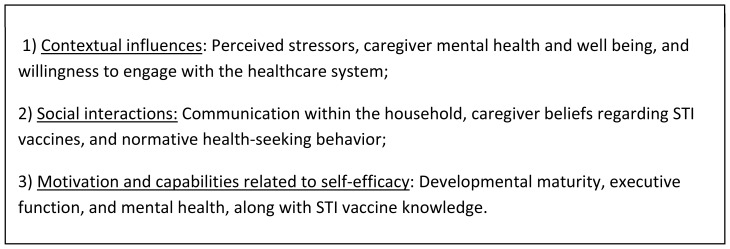
Topics of Qualitative Data Collection.

We recruited adolescents and their caregivers from the parent trial to participate in an in-depth semi-structured interview. We used a purposive sampling strategy for this study in order to tap multiple perspectives on decision-making related to HPV vaccine acceptability [37]. Trained qualitative researchers individually interviewed each study participant in their language of choice. The caregiver and adolescent were interviewed separately and confidentiality was maintained. Inquiry was guided by the domains from our prior conceptual framework [38]. Topics are shown in **Figure 1**. We used an iterative approach throughout the data collection process [39].

### Data Analyses

Interviews were digitally recorded, transcribed, translated as required, and entered into MAXQDA. Data analysis focused on characterizing reasons why adolescents and caregivers chose to initiate the HPV vaccine. Using an inductive approach based upon grounded theory, categories were constructed to name, define, and illustrate content themes [40]. We searched interview data for key concepts that pertained to vaccine-related decision-making, and displayed the text in matrices to identify patterns. Patterns of content appearing repeatedly in the data formed the basis for thematic categories. Once the key concepts were identified, three researchers developed a codebook (IK, JD and KP) [41]. This process continued iteratively until intercoder agreement exceeded 80%, indicating high levels of coder agreement [42]. Categories could be grouped into broad themes that provided an integrated explanation of why adolescents and their caregivers were eager to accept the HPV vaccine. These 4 broad themes are described in the Results section.

## Results

Two-hundred and twenty four adolescent participants were recruited in the parent trial at the Soweto study site, with 201 participants (89.7%) ultimately enrolling. Of these 201 participants who received the first dose of the vaccine, 192 (95.5%) returned for a second immunization at the 2-month visit. At the 6 month visit, 164 participants (81.6%) completed the full three doses of the vaccine. Characteristics of this population from the parent trial are presented in **Table 1**. Quantitative assessments were performed at all sites to assess understanding of the HPV vaccine at the initial screening after the consent process, prior to receiving the first dose of the vaccine. Mean scores for the checklist questions (n = 212) at recruitment at the Soweto site were: 11.7/13.

**Table 1:** Characteristics of Adolescents Presenting for HPV Vaccination in Soweto.

Sociodemographic Characteristics		Overall N = 201 (enrolled)
Age (years)	12–14	141 (70.1%)
	15–18+	60 (29.9%)
Race/Ethnicity	Black African	156 (77.6%)
	Colored/Other	45(22.4%)
Education level to date	Grade 10	13 (6.5%)
	Grade 9	26 (12.9%)
	Grade 8	36 (17.9%)
	<Grade 8	126 (62.7%)
Gender	Female Male	129 (64.2%) 72 (35.8%)

For our qualitative study, we chose a sub-population of individuals to interview to understand factors driving vaccine uptake. Forty-eight adults were approached, and ultimately, 39 eligible adolescent-caregiver dyads agreed to participate (n = 77). Over 60% of our 39 adolescent participants were girls and roughly 40% were boys; ranging in age from 12 to 19 years. All adolescents lived within walking distance to the Kganya Motshe Adolescent Clinic in the Kliptown section of Soweto. Most participants lived in informal settlements, which consisted of one to two room dwellings with tin roofs and cement floors. All 38 caregivers were women ranging in age between 34 and 61, and all but 2 were single. While we sought to include perspectives of participants along a continuum of decision-making, we were only able to obtain consent from adolescents and their caregivers who chose to participate in this trial and therefore accept the vaccine. Four prominent thematic influences on vaccine-related uptake were inductively identified through data analysis. These themes are described in detail below:

### 1) Single-headed Households Leading to Adolescent Autonomy in Health Decision-making

No male caregivers were identified in our sample. Women often self-identified as the primary caretakers within the household. They discussed feelings of hopelessness and an inability to keep their children safe, particularly as single mothers without a steady source of income. One mother described her concern that her son would “start doing bad things at an early stage,” because there was “no man in this house.” The caregivers repeatedly expressed wanting a “better life” for their children. This ultimately acted as a strong motivator to get children vaccinated to prevent STIs to “help our kids to do better.” Women also expressed a hope that STI vaccination would prevent their children from becoming sexually active:

*“It is a good thing for our kids because going to the clinic and getting the vaccine…it helps our kids not to do funny things. Since you can see how kids are these days. They have sex at an early age and end up being pregnant and things like that. But now, Kganya Motsha will help us keep our kids safe, because when my daughter goes for her visit, she will be checked all the time that she is still a virgin. So, I see it as something that is going to help me a lot.” – 47 year old mother of a 14 year old girl*

Adolescents described their caregivers as overburdened, reporting that mothers were often “stressed” and could “become cheeky” if topics related to sex were raised. Mothers were described as absent if they were employed. This often translated into adolescents feeling responsible for ensuring they remain “safe” and avoid early pregnancy or acquire a STI. In this context, adolescents we interviewed exercised a high level of autonomy in health decision-making, by going to the clinic alone to find out about the HPV vaccine trial, and then discussing it with their caregivers. Caregiver consent was often seen as a formality by the adolescent. One 14 year old girl mentioned that her mother agreed she could get the vaccine, but she had “already decided she was going to take it.” Adolescents often had to educate their caregivers about the benefits of vaccination, in order to get their consent to receive the HPV vaccine:

*“My mother didn’t want me to go and get the vaccine because she thought that maybe they will give us AIDS or something. So then I had to explain to her…and she understood that I wanted to go.” – 16 year old girl*

### 2) Role of Healthcare Providers and Peers in Influencing Vaccine Uptake and Providing Support

Caregivers expressed difficulty initiating conversations with their adolescents related to sexual activity or sexual risk-taking, due to lack of personal information and education. Healthcare providers at the adolescent clinic were described as adult proxies who could have conversations with their children about sexual health, in order to “provide knowledge about sexual issues.” Caregivers described the clinic as a refuge for their children, providing support and stability that was often unattainable in a chaotic household:

*“My son used to move with a bad group and I thought [enrolling him in the HPV vaccine trial] would help take him out off the street, so that he would have something to do after school. It would also help him, so that when he has questions from school, and I can’t help him, he can go to the sisters [who work at the clinic] for help.” – 33 year old mother of 16 year old boy*

Both caregivers and adolescents discussed the importance of peers in decision-making related to STI prevention. In general, adults felt that peers provided misinformation to their children, and encouraged each other to practice risky behavior. While adolescents acknowledged that peers often had “naughty behavior” that was “careless,” they tended to focus on the positive impact of peer-influence, particularly in regards to encouraging vaccine initiation.

*“I first went to the clinic. Then my other friend asked me what I was doing up there. I told him to come and that he would see what everything was about. He came and they made files for both of us and now we are both going to get the vaccine.” – 13 year old boy*

### 3) STI Vaccination as Harm-reduction Strategy in the Setting of Endemic Gender Based Violence

Caregivers perceived that adolescents started becoming sexually active in their early teens. They reported, however, that boys and girls were likely to become sexually active for different reasons. Caregivers of girls described sexual violence as being omnipresent, and adults often felt powerless to protect their children. Adults often suggested that they were highly motivated to get their daughters vaccinated against an STI due to a fear of sexual violence and age-discordant relationships that left girls uniquely vulnerable. Harm-reduction terminology was used when discussing the need for young girls to get vaccinated, with mothers often discussing the inevitability of rape, while hoping their daughters would be “protected” against acquisition of a STI. In this way, vaccination was perceived as a way to take control over a situation where they generally felt powerless:

*“I feel certain about [vaccinating my child] because there is AIDS and HIV out there and we all are aware of it. My child can be raped, and I will feel bad about it, but I am at peace [knowing] that she is participating in the Kganya Motsha [HPV vaccine trial] and will be protected against sexually transmitted diseases.”– Mother of a 14 year-old girl*

Young people corroborated these views, recognizing that friends in their age group were sexually active, but that this sexual activity was often in the form of sexual abuse or was performed in exchange for money, particularly in the context of an age-discordant relationship, with older men and younger girls. Adolescents described friends or neighbors who were raped, and others who would “sell themselves just to get money.”

### 4) Influence of the HIV Epidemic on Understanding of HPV Vaccine

All caregivers and adolescents discussed the omnipresence of HIV in their lives. Adolescents as young as 16 used terminology such as “monitoring a CD4 count,” and knew the difference between the HIV virus and an AIDS diagnosis.

*“HIV is a virus that causes AIDS. HIV doesn’t kill anybody. It is AIDS that kills a human being. AIDS is when the CD4 count, the white blood cells, goes down. So if it happens that the person’s white blood cells are weak it is easy for other diseases to get him or her and then that person will die fast.” – 16 year old girl*

This knowledge often came at the exclusion of knowing about any other STIs, with adolescents stating they only knew that HIV and AIDS could be acquired through sexual contact, and did not know “any other diseases that come through sex.” Despite a basic understanding of HPV, as demonstrated through the quantitative testing performed at the beginning of the parent trial, both adolescents and their caregivers expressed uncertainty in our interviews about whether the HPV vaccine was also potentially protective against HIV. Caregivers generally focused on HIV-prevention, discussing that HIV was “a death sentence,” and that vaccination against an STI was the only way for their children to remain safe.

*“The thing that really got me interested in this vaccine is because our youth are dying out with HIV and AIDS, and to me as a parent, I am very, very, concerned. It is very painful to see the children. They can’t bury us anymore. We live now to bury them, because of this disease.” –52 year old mother of a 15 year old boy*

## Discussion

In this qualitative study, we examined adolescents’ and caregivers’ views of HPV vaccination in order to understand factors influencing vaccine uptake in Soweto, South Africa. We found that factors driving vaccine uptake reflected a socio-cultural backdrop of high HIV endemnicity, sexual violence, poverty, and an abundance of female-headed households. Within this context, adolescents exercised a high level of autonomy, and often initiated decision-making. These qualitative findings build on prior research performed in South Africa related to HPV vaccine acceptability [33], [43], [44], and provide a context for understanding how adolescents and caregivers view the HPV vaccine, establishing a framework for optimizing programs that would introduce the HPV vaccine into the public health sector in South Africa [19].

While prior studies have focused on caregivers being the primary decision-makers regarding youth vaccination [45], [46], we found that most decisions made relating to HPV vaccine uptake were youth-driven. Prior research in South Africa has described the “feminization of poverty” as a situation in which young poor women, acting as the head of the household, are at unique risk [47]. Our data show that in this setting, adolescents are highly self-reliant and exercise great autonomy in decision-making, ultimately leading them to play a significant role in choosing whether or not to accept an STI vaccine. These findings shed light on the importance of understanding developmental factors within this larger social context of adolescent-autonomy, and the need to develop programs involving healthcare providers to engage young people in effective decision-making strategies.

This study also provided a unique perspective among adolescent participants, specifically the involvement of boys. While most qualitative research in resource-limited settings related to HPV vaccine uptake has focused on girls, prior findings in resource-rich settings have been performed with boys and men. Results from those studies show that boys may mistakenly posit that HPV only affects girls [48]. Despite this, boys have been shown to be important partners in decision-making [49], and quantitative studies have confirmed that boys are amenable to receiving the HPV vaccine [50], particularly if it is offered in conjunction with other recommended adolescent vaccines [51]. Our research furthers these findings by demonstrating that boys can be partners in decision-making (even, at times, taking the lead), and that it is feasible to administer a vaccine to an adolescent, even in the absence of an infrastructure to vaccinate young adults.

Our finding related to the fear of gender-based violence and age discordant relationships has been seen as a driver of vaccine uptake previously in South Africa [32], [33], [52], [53]. South Africa is reported to have one of the highest rates of sexual assault in the world, with girls between the ages of 12–15 at highest risk [54], . Fear of becoming a victim of sexual violence is commonly held in South Africa. In the most recent “Victims of Crime” survey, published by the South African Government [59], nearly a third (27.1%) of households feared sexual violence (including rape) more than other crimes in their area. While caregivers expressed deep concern about the level of sexual violence in their community, our data support the use of a “harm reduction” strategy as a way to protect young girls from STI acquisition while recognizing the reality of gender-based violence in Soweto.

Finally, when considering the term “vaccine acceptability,” we need to recognize that participants expressed a limited understanding of the HPV vaccine, despite careful counseling. While these findings mirror prior results in South Africa [33], [44], our study adds a unique perspective by specifically providing evidence of real-time HPV vaccine uptake despite this misconception. Confusion regarding HPV and HIV may reflect the fact that this trial used the HPV vaccine as a proxy for future HIV vaccination among adolescents, as well as the larger context of post-apartheid South Africa, where HIV has remained at epidemic levels [60]. Knowledge of HPV and its associated diseases has been found to be low [33], despite cervical cancer being the leading cancer-associated cause of death among South African women [12], [44]. Despite this, we believe that our findings show that this population of adolescents and their caregivers are highly motivated to seek interventions to help prevent the transmission of STIs. These interventions carry some degree of burden (specifically returning for a vaccine three times over the course of six months). This is novel, especially given the lack of adolescent vaccination programs in place in South Africa at this time.

This study had the notable limitation of being performed in the context of an observational trial. Participants who are part of a trial may be more likely to trust clinicians, which itself may be correlated with vaccine uptake. This phenomenon has been considered in other HPV trials [61]. In addition, the HPV vaccine was provided at no-cost for participants in this study, thereby enriching our sample of HPV vaccine accepters for adolescents and caregivers from low-income households. In addition, our study is exploratory in nature, and focused on identifying thematic influences on vaccine uptake and understanding relationships between these concepts without assigning a relative importance to these themes. Therefore, our findings cannot be generalizable to a larger population of adolescents and their caregivers. Rather, these findings provide an in-depth understanding of a specific context occurring in Soweto. Finally, given that fears of sexual violence, and experience of sexual violence itself, are associated with poverty [62], [63], our findings may have over-emphasized its relative importance in decision-making. Despite these limitations, these findings support prior research done at this site in Soweto where adolescents and their caregivers were eager to participate in biomedical prevention trials, and supports the translation of this willingness to participate to actual health-seeking behavior [64].

## Conclusions

Our findings provide an initial framework to understand STI vaccine uptake in a setting such as Soweto with epidemic levels of HIV, sexual violence, poverty, and an abundance of female-headed households. Future interventions should specifically target adolescents in female-headed households to assess acceptability and feasibility of STI primary-prevention strategies, while providing extensive education to adolescents and their caregivers about HPV, as well as other modalities of cervical cancer prevention.
